# Sustained outcomes 3 years after all-oral 9-month regimen for rifampicin-resistant TB

**DOI:** 10.5588/ijtldopen.25.0494

**Published:** 2026-02-11

**Authors:** T.M.P. Nguyen, T.H.M. Le, C.S.C. Merle, L. Guglielmetti, N.L. Nguyen, V.L. Dinh, B.H. Nguyen, T.T.T. Hoang, V.N. Nguyen, S. Callens, T. Decroo

**Affiliations:** 1 National Lung Hospital, Hanoi, Vietnam;; 2 Department of Internal Medicine and Infectious Diseases, Ghent University Hospital, Ghent, Belgium;; 3 Institute of Tropical Medicine Antwerp, Antwerp, Belgium;; 4 The Special Programme for Research and Training in Tropical Diseases (TDR), World Health Organization, Geneva, Switzerland;; 5 Department of Infectious, Tropical Diseases and Microbiology, IRCCS Sacro Cuore Don Calabria Hospital, Verona, Italy;; 6 Global Programme on Tuberculosis and Lung Health, World Health Organization, Geneva, Switzerland;; 7 Hanoi Medical University, Hanoi, Vietnam;; 8 Vietnam National University Ha Noi, University of Medicine and Pharmacy, Hanoi, Vietnam.

**Keywords:** tuberculosis, Vietnam, drug-resistant TB, shorter regimen

Dear Editor,

TB remains one of the most common life-threatening infectious diseases worldwide. Vietnam is one of the high TB and rifampicin-resistant TB (RR-TB) burden countries in the world, with an estimated 182,000 new TB cases in 2023. This includes about 9,900 RR-TB cases, of which only 36% received treatment in the same year.^1^ In 2025, the WHO recommended different regimens for RR-TB patients without confirmed fluoroquinolones (FQ) resistance, one of which is a 5-drug 9-month treatment regimen, based on results of the endTB clinical trial. This so called BCLLfxZ regimen includes bedaquiline (BDQ or B), clofazimine (CFZ or C), linezolid (LZD or L), levofloxacin (Lfx), and pyrazinamide (PZA or Z).^2–4^ In Vietnam, between 2020 and 2021, 106 patients were enrolled on BCLLfxZ. Interim analysis of this study demonstrated high (89.6%; 95/106) end-of-treatment success.^5^ Besides the 17-month endTB outcome data,^3^ there is no evidence on post-treatment outcomes for this regimen. We therefore complement our previously published results^5^ by showing long-term outcomes of BCLLfxZ regimen, until month 45 following treatment initiation (or the 36th post-treatment month). In addition, we show the final outcome of patients with treatment failure or a new TB episode after BCLLfxZ treatment completion.

In this prospective cohort study, conducted at six large treatment sites in Vietnam, 106 RR-TB patients without proof of initial FQ resistance (71 with FQ-susceptible TB, 35 without drug susceptibility testing [DST] result) were enrolled on the BCLLfxZ regimen and followed up until 36 months post-treatment. RR-TB was diagnosed via Xpert MTB/RIF (Cepheid Inc., USA), followed by FQ resistance screening using genotypic (GenoType MTBDRsl, Hain Lifescience, Germany) or phenotypic second-line DST. Exclusion criteria included prior exposure to second-line drugs (≥1 month), QTcF >500 ms, extra-pulmonary TB, severe electrolyte abnormality, end-stage liver or kidney disease, and pregnancy or breastfeeding. Eligible RR-TB patients started the BCLLfxZ regimen for 9–11 months (11 months if positive on culture or lack of clinical progression at month 4). After completing treatment, patients were scheduled for follow-up consultations at months 6 and 12 post-treatment, including clinical evaluation, smear, culture, and chest X-ray. A final assessment was conducted by treatment site’s nurses, by phone interviews at 36 months post-treatment. If symptoms were detected during the interview, participants were called for medical examinations and sputum collection.

Ethical approval was obtained from the National Lung Hospital in Vietnam. Written informed consent was obtained from all participants.

Of 106 patients, 95 (89.6%) achieved end-of-treatment success (cured or treatment completed), while 6 (5.7%) were lost to follow-up (LTFU), 1 (0.9%) died, and 4 (3.8%) experienced treatment failure: 3 due to permanent regimen changes after adverse events and 1 due to culture reversion (see Figure).^5^ Among the 95 successfully treated patients, 87.4% (83/95) had post-treatment follow-up data: 83.1% (69/95) until month 36, 10.9% (9/95) until month 12, and 6% (5/95) until month 6. Fourteen out of 95 patients reported TB-like symptoms at any time during follow-up. Of those, one patient had rifampicin-susceptible TB on Xpert MTB/RIF and completed treatment with a treatment regimen for drug-susceptible TB. This recurrence case was possibly re-infected with a susceptible strain. FQ or BDQ DST was not done considering the national algorithm for rifampicin-susceptible TB. TB recurrence was not confirmed among others. Thus, 3 years post-treatment, the BCLLfxZ regimen cohort achieved 88.7% (94/106) sustained success (cured or treatment completed without recurrence). The four patients with treatment failure during BCLLfxZ were switched to longer, individualised RR-TB retreatment regimens. Among three changing regimens due to adverse events, two were successfully treated and one was LTFU after culture conversion at treatment month 12. The patient with failure due to culture reversion on BCLLfxZ also experienced failure during retreatment and died. Overall, after accounting for BCLLfxZ and (RR-TB) retreatment outcomes, of 106 patients enrolled, 97 (91.5%) were cured/completed, 7 were LTFU, and 2 died.

**Figure.**
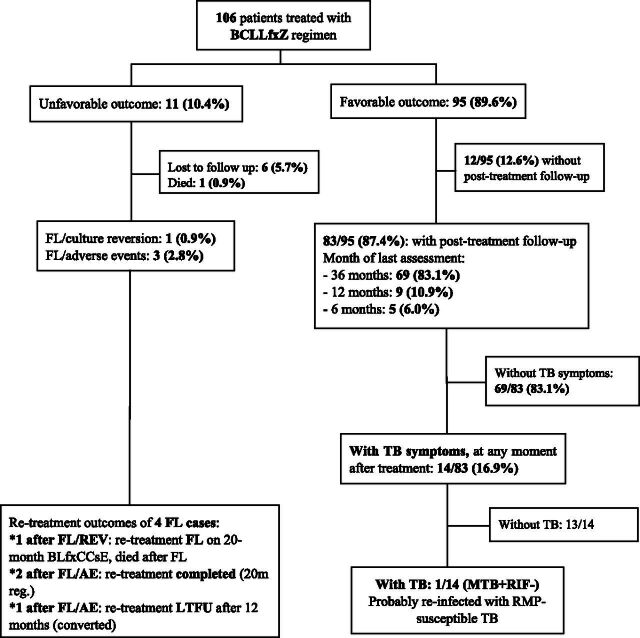
Post-treatment outcomes 3 years after RR-TB treatment by BCLLfxZ regimen in Vietnam. B = bedaquiline; C = clofazimine; L = linezolid; Lfx = levofloxacin; Z = pyrazinamide; Cs = cycloserine; E = ethambutol; FL = failure; FL/REV = failure/culture reversion; FL/AE = failure/adverse events; LTFU = lost to follow-up; MTB+/RIF− = *Mycobacterium tuberculosis* confirmed and not resistant to rifampicin; DST = drug susceptibility testing; 20 m reg. = 20-month regimen.

The five-drug all-oral BCLLfxZ regimen in our study showed high long-term treatment success. These results support the findings from the endTB trial, which reported 90.4% (104/115) sustained success at 17 months post-randomisation (8 months after treatment completion), with an extended follow-up period.^3^ Our results show long-term effectiveness of the WHO-recommended BCLLfxZ regimen under programmatic conditions,^2^ offering a robust short-course treatment option for FQ-susceptible RR-TB, particularly in settings where the 6-month BPaL(M) regimen is not yet available. Those not eligible for either short regimen will still require a long-duration regimen.^6^

In comparison with other studies of modified 9-month regimens (mSTR), our results are excellent. For instance, mSTR cohorts in the Dominican Republic (N = 113) and multiple European countries (N = 2,636), also using BDQ and FQ as core drugs, and LZD and CFZ as companion drugs,^7,8^ but with cycloserine instead of Z in the regimen, reported slightly lower recurrence-free success rates (∼79%) within 6–12 months post-treatment.^9,10^ While our cohort was smaller, the 36-month post-treatment follow-up provides valuable insights into the regimen’s sterilising capacity.^11,12^ This is critical, as shorter regimens may lead to bacteriological relapses.^13,14^

End-of-treatment success of about 90% of our simplified standardised 9-month regimen also compares favourably with 72% success reported in Vietnam’s overall national cohort,^1^ providing a viable choice for FQ-susceptible RR-TB patients in Vietnam. Although 25 patients (23.6%) discontinued Z during treatment due to baseline resistance identified after regimen initiation,^5^ no patient had recurrent RR-TB during 3 years of post-treatment follow-up, suggesting sufficient sterilising potential of the four remaining drugs. Whether a 4-drug regimen, including BDQ, FQ, LZD, and CFZ, can be as effective as the 5-drug BCLLfxZ regimen warrants further investigation.

Our study has several strengths. It employed a prospective design, with rigorous data collection. Long-term post-treatment follow-up adds evidence on relapse and recurrence following novel short-course regimens. We described what became of patients with treatment failure (N = 4) or a new episode of TB (N = 1), thus providing a more comprehensive view on final outcomes. Importantly, the study provides insights into the regimen’s effectiveness within routine programmatic care. Nevertheless, limitations of this study include the small sample size and incomplete post-treatment follow-up among some of the cured patients. Of the 95 patients with successful outcomes, 12.6% (N = 12) could not be contacted or assessed and 22.1% (N = 21) were only interviewed by phone. However, patients reporting TB-like symptoms during interviews were referred for clinical evaluation. Additionally, the national TB program is the sole provider of TB care in Vietnam, and recurrence not captured during scheduled follow-up would likely be identified during clinical evaluation in those with a new TB episode. Although treatment failure seems rare, retreatment may be very challenging, as shown by the outcome of the participant who did not respond to two courses of treatments and died. Contributing factors may be poor access to DST for novel second-line drugs, hampering the design of retreatment regimens.^15^

In conclusion, our study showed high and sustained treatment success of the 9-month BCLLfxZ regimen. Where the 6-month pretomanid-containing regimen is not yet available or in case of contraindications to pretomanid, BCLLfxZ is a robust alternative for patients with FQ-susceptible RR-TB.
